# Illinois

**Published:** 1898-06

**Authors:** 


*Illinois.*—During 1896–97 resignations of the following Assistant State Veterinarians were tendered and accepted by the board: Drs. Joseph Hughes, Chicago; A. S. Alexander, Evanston; J. F. Ryan, Chicago; H. G. Pyle, Springfield; George Ditewig, Canton; J. Stallman, Pontiac; R. C. Mylne, Aurora; A. H. Baker, Chicago; W. A. Baker,[1] Gibson City.

The following appointments of Assistant State Veterinarians were made by the State Veterinarian and advised by and consented to by the board: Drs. W. J. Thomas, Henry; W. J. Martin, Kankakee; S. Snyder, Fairfield; J. M. Wright, 1639 Wabash Avenue, Chicago; J. L. Tyler, Chebanse; B. F. Hoover, Davis; F. B. Rowan, Belvidere; S. Schoenleber, Morris; J. B. Walsh, Monticello, vice Frank Bales, Monticello.

The following is the revised list of Assistant State Veterinarians now holding commissions: W. E. McGrath, Union Stock Yards, Chicago; L. C. Tiffany, Springfield; J. A. T. Hill, Chicago Lawn,

[1] Removed from the State.

Chicago; A. McGuire, 1446 Wabash Avenue, Chicago; B. A. Pierce, Union Stock Yards, Chicago; C. A. Pierce, Elgin; B. F. Swingley, Freeport; J. J. Walker, Olney; F. W. Rowan, De Kalb; Charles W. Johnson, Elburn; J. F. Reed, Decatur; James Bond, Austin; S. V. Ramsey, Tuscola; A. J. Ziegler, Lincoln; B. B. Page, Rockford; John C. Stewart, Danville; J. D. Nighbert, Pittsfield; F. H. Armstrong, East St. Louis; Walter Tomlinson, Alexis; J. F. Pease, Quincy; O. J. McGurty, Paris; A. G. Alverson, Bloomington; Matthew Wilson, Mendota; C. M. Paxton, Kansas; P. C. Dodge, Rochelle; W. F. Weese, Ottawa; James Addison, Aledo; Thomas Hope, Waukegan; J. C. Booker, Alton; I. J. Miles, Charleston; John Scott, Peoria; W. J. Lawson, Petersburg; H. G. Hoover, Sterling; L. F. Brown, Galesburg; E. S. Fry, Naperville; G. L. Bauer, Belleville; C. C. Behner, Marshall; W. J. Thomas, Henry; W. J. Martin, Kankakee; S. Snyder, Fairfield; J. M. Wright, 1639 Wabash Avenue, Chicago; J. L. Tyler, Chebanse; B. F. Hoover, Davis; F. B. Rowan, Belvidere; S. Schoenleber, Morris; J. B. Walsh, Monticello.

During the past year applications for testing thirty-six herds were received, numbering some seven hundred animals, of which seventy-seven responded, and all proved tuberculous on autopsy; of this number forty-one were condemned as unfit for food, and thirty-six were passed, the disease in the latter being very limited and localized. The State Live Stock Sanitary Board recommends the enactment of a law compelling the owners of dairy-cattle who are selling milk for human consumption or to creameries, and the owners of breeding herds within a reasonable time, to be designated in the act, to submit their cattle to a tuberculin-test under the supervision of this board, providing that the cattle afterward introduced into the tested herds shall be first tested with tuberculin; that all cattle responding to the test sufficiently, in the judgment of the board to indicate the presence of tuberculosis in the animals, shall be slaughtered and the carcass disposed of as the board shall direct; that the owner in such cases shall receive a stipulated compensation, in part, at least, remunerating him for the loss sustained; providing further for retesting all such herds at such times as the board shall deem necessary to thoroughly eradicate the disease, and that the necessary appropriation be made to carry out the provisions of such an act.

